# Cancer incidence after asthma diagnosis: Evidence from a large clinical research network in the United States

**DOI:** 10.1002/cam4.5875

**Published:** 2023-03-31

**Authors:** Yi Guo, Jiang Bian, Zhaoyi Chen, Jennifer N. Fishe, Dongyu Zhang, Dejana Braithwaite, Thomas J. George, Elizabeth A. Shenkman, Jonathan D. Licht

**Affiliations:** ^1^ Department of Health Outcomes and Biomedical Informatics, College of Medicine University of Florida Gainesville Florida USA; ^2^ University of Florida Health Cancer Center Gainesville Florida USA; ^3^ Department of Emergency Medicine, College of Medicine – Jacksonville University of Florida Jacksonville Florida USA; ^4^ Department of Epidemiology University of Florida Gainesville Florida USA; ^5^ Division of Hematology and Oncology, Department of Medicine, College of Medicine University of Florida Gainesville Florida USA

**Keywords:** electronic health records, inhaled steroid, prediction, real‐word data, survival analysis

## Abstract

**Background:**

Prior studies on the association between asthma and cancer show inconsistent results. This study aimed to generate additional evidence on the association between asthma and cancer, both overall, and by cancer type, in the United States.

**Method:**

We conducted a retrospective cohort study using 2012–2020 electronic health records and claims data in the OneFlorida+ clinical research network. Our study population included a cohort of adult patients with asthma (*n* = 90,021) and a matching cohort of adult patients without asthma (*n* = 270,063). We built Cox proportional hazards models to examine the association between asthma diagnosis and subsequent cancer risk.

**Results:**

Our results showed that asthma patients were more likely to develop cancer compared to patients without asthma in multivariable analysis (hazard ratio [HR] = 1.36, 99% confidence interval [CI] = 1.29–1.44). Elevated cancer risk was observed in asthma patients without (HR = 1.60; 99% CI: 1.50–1.71) or with (HR = 1.11; 99% CI: 1.03–1.21) inhaled steroid use. However, in analyses of specific cancer types, cancer risk was elevated for nine of 13 cancers in asthma patients without inhaled steroid use but only for two of 13 cancers in asthma patients with inhaled steroid use, suggesting a protective effect of inhaled steroid use on cancer.

**Conclusion:**

This is the first study to report a positive association between asthma and overall cancer risk in the US population. More in‐depth studies using real‐word data are needed to further explore the causal mechanisms of asthma on cancer risk.

## Graphical abstract

Adjusting for the covariates, patients with asthma were 1.36 times as likely to develop cancer compared to patients without asthma (Hazard Ratio [HR] = 1.36; 99% Confidence Interval [CI]: 1.29–1.44). Compared to patients without asthma, elevated cancer risk was observed in asthma patients without (HR = 1.60; 99% CI: 1.50–1.71) or with (HR = 1.11; 99% CI: 1.03–1.21) inhaled steroid use.

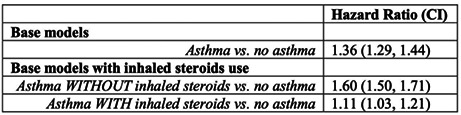

## 1 INTRODUCTION

As the second leading cause of death, cancer is responsible for one in every four deaths in the United States.^1^ One of the primary research areas in cancer etiology has been the relationship between chronic inflammation and cancer.^2^, ^3^ Prior research suggests that about 25% of all cancer cases may be caused by chronic infection and inflammation.^3^ Inflammation usually starts from an infection or a tissue injury, to which the innate and adaptive immune systems provide a series of well‐coordinated responses.^2^, ^3^ However, failure in the precise control of acute inflammatory responses can lead to chronic inflammation, which creates a pathological microenvironment conducive to cancer initiation and progression. Likely as a result, the risk of developing cancer is elevated in patients with chronic inflammatory diseases.^2^, ^3^

Asthma is a common condition in which complex and chronic inflammation is heavily implicated.^4^ As one of the most frequently diagnosed respiratory diseases, asthma affects over 20 million adults (8%) and over 5 million children (7%) in the United States.^5^ Due to the potential oncogenic effect of asthmatic inflammation, numerous studies have examined the association between asthma and the risk of developing cancer in different populations. For example, a 2017 meta‐analysis found that patients with asthma were 44% more likely to develop lung cancer than patients without asthma.^6^ However, other prior studies showed inconsistent results on the association between asthma and cancers other than lung cancer, reporting positive associations,^7^, ^8^, ^9^, ^10^, ^11^, ^12^ negative associations,^7^, ^8^, ^10^, ^11^, ^13^, ^14^, ^15^, ^16^, ^17^ or no association.^11^, ^18^

In addition, most prior studies on the relationship between asthma and cancer focused on European^7^, ^8^, ^11^, ^16^ or East Asian^9^, ^10^, ^12^, ^17^ populations, or on a single cancer type such as colorectal^13^, ^14^ or head and neck cancer.^15^ To date, only one study has examined the association between asthma and the risk of developing cancer overall, in addition to specific types of cancers in the US population.^18^ In that study, Kantor et al. analyzed survey and cancer registry data on residents of southern states aged 40–79 years and found no association between a history of asthma and subsequent risk of cancer, except for lung cancer.^18^ Considering the scarcity of evidence linking asthma and cancer in the US population as well as the inconsistent findings in other populations, we searched for an association between asthma and cancer using the OneFlorida+ Clinical Research Network (CRN), a large database of real‐world data, including electronic health records (EHRs) and claims data.^19^ We also considered the modifying effect of inhaled steroids on asthma in our analyses, which have been shown to alter the risk of developing lung cancer in some asthma patients.^20^

## 2 METHODS

### 2.1 Study design and study population

This was a retrospective cohort study using 2012–2020 EHR and claims data from the OneFlorida+ CRN,^19^ one of nine CRNs in the national PCORnet funded by the Patient‐Centered Outcomes Research Institute. OneFlorida+ contains robust longitudinal, linked, patient‐level real‐word data for over 19 million patients, including data from EHRs, Medicaid claims, cancer registries, and vital statistics.

Our study population included a cohort of patients with a first asthma diagnosis and a matching cohort of adult patients without asthma in OneFlorida+. We included patients aged 18–65 years who had at least one inpatient or two outpatient visits each year from 2012 through the end of 2015. Next, we identified asthma patients using the International Classification of Diseases (ICD) codes for asthma (ICD‐9: 493; ICD‐10: J45). An asthma diagnosis was confirmed if a patient had at least one inpatient or two outpatient asthma diagnoses within 1 year. We defined the index date as the date of the first asthma diagnosis for the asthma patients. Asthma patients who had any cancer diagnosis (ICD‐9: 140–209; ICD‐10: C00–C96) before or 30 days after the index date were excluded from our analysis.

To create a matching cohort of non‐asthma patients as the comparison group, we used the frequency matching method to match each asthma patient with three randomly selected non‐asthma patients based on age and sex. The non‐asthma patients must have at least one inpatient or outpatient encounter within 1 year of the asthma patient's index date. The index date for the non‐asthma patients was defined as the date of their encounter closest to the matched asthma patient's index date. Similarly, non‐asthma patients who had any cancer diagnosis (ICD‐9: 140–209; ICD‐10: C00–C96) before or 30 days after the index date were also excluded from our analysis.

### 2.2 Primary exposure and outcome

The primary exposure was asthma diagnosis. The primary outcome was time to incident cancer diagnosis after the index date. We used ICD codes to identify all cancer patients (ICD‐9: 140–209; ICD‐10: C00–C96) and patients with cancers of the female breast (ICD‐9: 174; ICD‐10: C50), prostate (ICD‐9: 185; ICD‐10: C61), lung (ICD‐9: 162; ICD‐10: C34), colorectal (ICD‐9: 153, 154, 159.01; ICD‐10: C18–C21, C26.0), blood (ICD‐9: 200–208; C81–C96), melanoma (ICD‐9: 172, 173; ICD‐10: C43, C44), endometrial (ICD‐9: 182; ICD‐10: C54), bladder (ICD‐9: 188; ICD‐10: C67), kidney (ICD‐9: 189; ICD‐10: C64), oral cavity and pharynx (ICD‐9: 140–149; ICD‐10: C00–C14), pancreas (ICD‐9: 157; ICD‐10: C25), ovarian (ICD‐9: 183; ICD‐10: C56), and cervix (ICD‐9: 180; ICD‐10: C53).

### 2.3 Covariates

For all patients in the study population, we obtained information on the following covariates in EHRs: age, sex, race/ethnicity, smoking status, systolic blood pressure (SBP), diastolic blood pressure (DBP), Charlson comorbidity index (CCI), and history of diabetes mellitus, chronic obstructive pulmonary disease (COPD), stroke, and hypertension. Age, sex, and race/ethnicity were determined at the index date. Smoking status (current, former, or never smoker), SBP, and DBP were determined using the most recent EHR data for those variables before the index date. CCI was calculated using the Deyo's formula based on all records before the index date and categorized into three groups (0, 1, or 2 or more).^21^

### 2.4 Data analysis

Cox proportional hazards regression models were built to examine the association between asthma and subsequent cancer diagnoses. For all Cox models, the outcome was time (in months) from index date to the first cancer diagnosis and the primary predictor was asthma diagnosis (i.e., asthma or no asthma). Patients who did not develop any cancer were censored at the date of last encounter in EHRs. Separate Cox models (i.e., base models) were constructed for all cancers (i.e., any cancer) and each included cancer type. Model covariates included age, sex, race/ethnicity, smoking status, SBP, DBP, CCI, and history of COPD, diabetes mellitus, stroke, and hypertension. In addition, we examined whether inhaled steroid use modified the effect of asthma on cancer risk. To do so, we re‐built all Cox models (i.e., base models considering inhaled steroids use) with a modified asthma status variable as the primary predictor (i.e., asthma with inhaled steroid use, asthma without inhaled steroid use, or no asthma). To determine inhaled steroid use, we examined the patients' prescribing and dispensing records in the EHRs for any records of beclomethasone, budesonide, fluticasone, salmeterol, or mometasone.^22^ To control for multiple testing, we used the Bonferroni correction and performed all tests at 0.0038 significance level (0.05 divided by 13 cancers) and reported 99% confidence intervals (CIs) for hazard ratios (HRs). We evaluated the proportional hazards assumption by testing the significance of time‐dependent covariates in models and visually examining log (−log) survival curves for parallelism. We also reported the *c*‐statistic as a measure of goodness of fit for each model. The proportional hazards assumption was satisfied for all models, and all models showed good to excellent model fit (Table 2). All data analyses were performed using Python or R.

## 3 RESULTS

The patients' demographic and clinical characteristics were summarized in Table 1. Overall, we identified 90,021 asthma patients and 270,063 matched non‐asthma patients in OneFlorida+ between 2012 and 2015. The asthma patients and the matched non‐asthma patients had comparable distributions for the matching variables age (*p* = 0.999) and sex (*p* = 0.999). The average age was 39.3 years (standard deviation = 14.1 years) for the asthma patients and 39.7 years (standard deviation = 13.8 years) for the non‐asthma patients. The majority of patients were women (69.5% and 70.8% in asthma and non‐asthma patients, respectively) and white (50.3% and 44.5% in asthma and non‐asthma patients, respectively). Compared to the non‐asthma patients, the asthma patients had a higher rate of current smoking (27.2% vs. 18.6%; *p* < 0.001), COPD (9.6% vs. 1.8%; *p* < 0.001), stroke (1.7% vs. 1.2%; *p* = 0.002), hypertension (18.4% vs. 9.9%; *p* < 0.001), and diabetes (7.0% vs. 5.9%; *p* < 0.001). Overall, a higher percentage of asthma patients were diagnosed with cancer following the index date than non‐asthma patients (2.9% vs. 1.8%; *p* < 0.001). The average follow‐up time was 13.3 and 14.8 months for the asthma and non‐asthma patients, respectively.

**TABLE 1:** Patients' demographic and clinical characteristics.

	Asthma patients (*N* = 90,021)	Non‐asthma patients (*N* = 270,063)	*p*‐Value
	Mean (SD) or *n* (%)	Mean (SD) or *n* (%)	
Age	39.3 (14.1)	39.7 (13.8)	0.999
Sex			0.999
Women	62,540 (69.5%)	191,117 (70.8%)	
Men	27,481 (30.5%)	78,946 (29.2%)	
Race/ethnicity			<0.001
Non‐Hispanic White	45,291 (50.3%)	120,182 (44.5%)	
Non‐Hispanic Black	25,105 (27.9%)	62,773 (23.2%)	
Hispanic	15,328 (17.0%)	63,399 (23.5%)	
Other	4297 (4.8%)	23,709 (8.8%)	
CCI category			<0.001
0	60,323 (67.0%)	195,367 (72.3%)	
1	16,843 (18.7%)	47,924 (17.7%)	
≥2	12,855 (14.3%)	26,772 (9.9%)	
Smoking status			<0.001
Current	24,480 (27.2%)	50,358 (18.6%)	
Former	19,242 (21.4%)	41,520 (15.4%)	
Never	46,299 (51.4%)	178,185 (66.0%)	
Diastolic blood pressure	74.7 (8.86)	75.9 (13.2)	<0.001
Systolic blood pressure	125 (12.9)	127 (18.8)	<0.001
Having Diabetes	6299 (7.0%)	15,860 (5.9%)	<0.001
Having COPD	8687 (9.6%)	4983 (1.8%)	<0.001
Having Stroke	1568 (1.7%)	3348 (1.2%)	0.021
Having Hypertension	16,601 (18.4%)	26,807 (9.9%)	<0.001
Any cancer	2635 (2.9%)	4982 (1.8%)	<0.001

Abbreviations: CCI, Charlson comorbidity index; COPD, chronic obstructive pulmonary disease; SD, standard deviation.

The adjusted HRs from the Cox regression base models were summarized in Table 2. Adjusting for the covariates, patients with asthma were 1.36 times as likely to develop cancer compared to patients without asthma (HR = 1.36; 99% CI: 1.29–1.44). For the specific cancer types analyzed, elevated cancer risk was observed in asthma patients for five of the 13 cancers, including cancer of the lung (HR = 1.56; 99% CI: 1.33–1.83), blood (HR = 1.26; 99% CI: 1.08–1.47), melanoma (HR = 1.98; 99% CI: 1.67–2.36), kidney (HR = 1.48; 99% CI: 1.11–1.99), and ovary (HR = 1.88; 99% CI: 1.40–2.51).

**TABLE 2:** Adjusted hazard ratios for asthma status in Cox models for time to cancer occurrence.

Cancer type	Base models^a^		Base models considering inhaled steroids use^a^		
	Asthma (*n* = 90,021) vs. no asthma (*n* = 270,063)		Asthma without inhaled steroids use (*n* = 59,865) vs. no asthma (*n* = 270,063)	Asthma with inhaled steroids use (*n* = 30,156) vs. no asthma (*n* = 270,063)	
	Adjusted HR (99% CI)	*c*‐statistic	Adjusted HR (99% CI)	Adjusted HR (99% CI)	*c*‐statistic
All cancers	1.36 (1.29, 1.44) *	0.726	1.60 (1.50, 1.71) *	1.11 (1.03, 1.21) *	0.752
Breast	1.14 (0.93, 1.41)	0.836	1.18 (0.91, 1.52)	1.10 (0.84, 1.45)	0.804
Prostate	1.09 (0.85, 1.40)	0.949	1.50 (1.13, 1.99) *	0.68 (0.46, 1.00)	0.947
Lung	1.56 (1.33, 1.83) *	0.843	1.74 (1.44, 2.11) *	1.39 (1.14, 1.70) *	0.896
Colorectum	1.15 (0.95, 1.39)	0.732	1.51 (1.21, 1.88) *	0.77 (0.58, 1.03)	0.741
Blood	1.26 (1.08, 1.47) *	0.724	1.44 (1.21, 1.72) *	1.05 (0.85, 1.29)	0.783
Melanoma	1.98 (1.67, 2.36) *	0.826	2.05 (1.66, 2.52) *	1.92 (1.55, 2.38) *	0.817
Corpus uteri	1.13 (0.82, 1.54)	0.796	1.76 (1.26, 2.46) *	0.49 (0.28, 0.85) *	0.709
Bladder	0.91 (0.62, 1.33)	0.776	1.02 (0.63, 1.63)	0.80 (0.48, 1.34)	0.769
Kidney	1.48 (1.11, 1.99) *	0.754	1.52 (1.06, 2.17) *	1.45 (0.99, 2.11)	0.736
Oral cavity and pharynx	1.15 (0.86, 1.54)	0.797	1.31 (0.92, 1.85)	0.99 (0.67, 1.47)	0.783
Pancreas	1.03 (0.73, 1.45)	0.816	1.31 (0.88, 1.95)	0.74 (0.44, 1.22)	0.812
Ovary	1.88 (1.40, 2.51) *	0.815	2.31 (1.66, 3.21) *	1.41 (0.94, 2.10)	0.895
Cervix	1.15 (0.87, 1.51)	0.809	1.46 (1.08, 1.98) *	0.77 (0.51, 1.16)	0.855

Abbreviations: CI, confidence interval; HR, hazard ratio.

^a^ All models adjusted for age, sex, race/ethnicity, smoking status, Charlson comorbidity index, blood pressures, and history of chronic obstructive pulmonary disease, diabetes mellitus, stroke, and hypertension.

* indicates the HR was statistically significant.

The adjusted HRs from the Cox regression base models considering inhaled steroid use were also summarized in Table 2. A total of 30,156 out of 90,021 (33.5%) asthma patients had used inhaled steroids. Compared to patients without asthma, elevated cancer risk was observed in asthma patients without (HR = 1.60; 99% CI: 1.50–1.71) or with (HR = 1.11; 99% CI: 1.03–1.21) inhaled steroid use. For the specific cancer types, elevated cancer risk was observed in asthma patients without inhaled steroid use for nine of the 13 cancers, including cancer of the prostate (HR = 1.50; 99% CI: 1.13–1.99), lung (HR = 1.74; 99% CI: 1.44–2.11), colorectum (HR = 1.51; 99% CI: 1.21–1.88), blood (HR = 1.44; 99% CI: 1.21–1.72), melanoma (HR = 2.05; 99% CI: 1.66–2.52), corpus uteri (HR = 1.76; 99% CI: 1.26–2.46), kidney (HR = 1.52; 99% CI: 1.06–2.17), ovary (HR = 2.31; 99% CI: 1.66–3.21), and cervix (HR = 1.46; 99% CI: 1.08–1.98). On the other hand, elevated cancer risk was observed in asthma patients with inhaled steroid use for only two of the 13 cancers, including cancer of the lung (HR = 1.39; 99% CI: 1.14–1.70) and melanoma (HR = 1.92; 99% CI: 1.55–2.38). Asthma patients who used inhaled steroids were less likely to develop cancer of the corpus uteri than patients without asthma (HR = 0.49; 99% CI: 0.28–0.85).

## 4 DISCUSSION

Using EHR and claims data from the OneFlorida+ CRN, we found that patients with asthma were 1.36 times as likely to develop cancer compared to patients without asthma in multivariable analysis. Overall, elevated cancer risk was observed in asthma patients with or without inhaled steroid use. However, as shown in multivariable models for specific cancer types, cancer risk was elevated for nine of the 13 cancers in asthma patients without inhaled steroid use but only for two of the 13 cancers in asthma patients with inhaled steroid use, suggesting a protective effect of inhaled steroid use on cancer.

Our finding of a positive relationship between asthma and cancer risk is consistent with a subset of prior studies on non‐US populations.^7^, ^8^, ^9^, ^10^, ^11^, ^12^ Two Swedish studies, one on hospitalized asthma patients and the other on primary care asthma patients,^7^, ^8^ reported an overall increased risk of cancer in these patients. In Asia, one study on the Taiwanese population found that the overall cancer risk was slightly elevated in female patients with asthma,^10^ while another study on South Korean adults found that having asthma was associated with a 75% increase in the risk of incident cancer overall.^12^ Regarding individual cancer sites, a German study found an increased risk of prostate, breast, lung and colorectal cancers in patients with atopic diseases including asthma.^11^ Another Taiwanese study reported a positive relationship between asthma and prostate cancer.^9^

On the other hand, results from our study do not appear to align well with those from the US studies on the relationship between asthma and cancer.^13^, ^14^, ^15^, ^18^ For example, Kantor et al. failed to find a significant association between asthma and cancer risk overall among residents of southern states aged between 40 and 79 years, with the exception of lung cancer risk in stratified analyses.^18^ However, Kantor et al. used self‐reported history of asthma collected in patient surveys for data analysis, whereas our study used combinations of ICD codes to identify asthma diagnosis in EHRs which is associated with much higher sensitivity and specificity. It is possible that noise or inconsistencies in survey data dependent on recollection masked or dwarfed the effect of asthma on cancer occurrence preventing the elucidation of a link. It is also possible that the demographic differences between patients in the OneFlorida+ EHR versus other US‐based studies may mean different baseline genetic risk factors for cancer. Furthermore, several other US studies implied a nonsignificant or negative association between asthma and the risk of colorectal or head and neck cancers.^13^, ^14^, ^15^ However all of those studies analyzed history of allergy as the primary exposure (e.g., asthma, hay fever, and other allergic conditions) rather than patients with asthma alone. Nonetheless, our finding of a positive association between asthma and cancer risk appears to be a first in the US population. The speculative nature of chronic inflammation (e.g., asthma as a common example) as a driver for pan‐cancer development requires more investigation. Specifically, more in‐depth studies, especially those using real‐world data, are needed to further confirm this association and elucidate biomarkers that may truly identify those at highest risk.

Prior studies have shown a protective effect of inhaled steroid use on some cancers,^20^, ^23^, ^24^, ^25^ potentially through reducing inflammation. For example, Wang et al. found that inhaled corticosteroids use was associated with a reduced risk of lung cancer and suggested that regular prescription of inhaled corticosteroids may decrease inflammation and reduce lung cancer risk.^20^ More clinical and epidemiologic studies are needed to further examine the relationship between inhaled steroids and cancer risk, and explore the mechanism behind it.

A main strength of our study is the use of real‐word data in a large CRN in the national PCORnet. The rich data in OneFlorida+ allowed us to accurately identify our study population of asthma patients, their detailed medical history, and cancer outcomes. Our study does have several limitations to note. First, due to the observational nature of the study, our results do not prove any causal relationship between asthma diagnosis and cancer occurrences. Second, for patients who developed multiple cancers, we considered the cancer diagnosed first as the primary cancer in data analysis which could lower the HR estimates. Third, we were unable to control for some potentially important covariates such as etiologically related variables (e.g., TNF‐alpha) or environmental exposures due to the limitations of the EHR data.

## 5 CONCLUSIONS

This is the first study to report a positive association between asthma and overall cancer risk in the US population. More in‐depth studies using real‐word data are needed to further explore the causal mechanisms of asthma on cancer risk.

## AUTHOR CONTRIBUTIONS

**Yi Guo:** Conceptualization (equal); formal analysis (lead); investigation (lead); methodology (lead); project administration (equal); resources (equal); writing – original draft (lead). **Jiang Bian:** Conceptualization (equal); data curation (lead); resources (lead); writing – review and editing (equal). **Zhaoyi Chen:** Formal analysis (supporting); investigation (supporting); methodology (supporting). **Jennifer Fishe:** Investigation (equal); writing – review and editing (equal). **Dongyu Zhang:** Investigation (equal); writing – review and editing (equal). **Dejana Braithwaite:** Investigation (equal); writing – review and editing (equal). **Thomas J George:** Investigation (equal); writing – review and editing (equal). **Elizabeth A. Shenkman:** Conceptualization (equal); investigation (equal); writing – review and editing (equal). **Jonathan D. Licht:** Conceptualization (equal); investigation (equal); writing – review and editing (equal).

## CONFLICT OF INTEREST STATEMENT

The authors have no conflicts of interest to disclose.

## ETHICS STATEMENT

This study was approved by the University of Florida Institutional Review Board.

## Data Availability

De‐identified data that support the findings of our study are available from the corresponding authors upon reasonable request.
